# Evaluation of the corneal epithelium in non-Sjögren’s and Sjögren’s dry eyes: an in vivo confocal microscopy study using HRT III RCM

**DOI:** 10.1186/s12886-018-0971-3

**Published:** 2018-12-04

**Authors:** Olivia L. Lee, Tudor C. Tepelus, Jianyan Huang, Anne G. Irvine, Christopher Irvine, Gloria B. Chiu, SriniVas R. Sadda

**Affiliations:** 1 Doheny Image Reading Center, Doheny Eye Institute, 1355 San Pablo Street, Los Angeles, CA 90033 USA; 2 Keck School of Medicine, Roski Eye Institute, 1450 San Pablo Street, Los Angeles, CA 90033 USA; 3 Universidad Autonoma de Guadalajara, Lomas del Valle, 45129 Zapopan, Jalisco Mexico; 4 Department of Ophthalmology, David Geffen School of Medicine at UCLA, Los Angeles, CA 91105 USA

**Keywords:** Dry eye syndrome, Sjögren’s syndrome, corneal epithelium, *in vivo* laser scanning confocal microscopy

## Abstract

**Background:**

The corneal epithelium is directly affected in dry eye syndrome. Thus, we attempted to describe the morphological features and evaluate the cellular density within the corneal epithelial layers in patients with non-Sjögren’s (NSDE) and Sjögren’s syndrome dry eyes (SSDE) by in vivo confocal microscopy (IVCM).

**Methods:**

Central cornea was prospectively imaged by IVCM in 68 clinically diagnosed aqueous tear-deficient dry eyes and 10 healthy age-matched control eyes. Morphological characteristics of corneal epithelial layers and cellular densities were evaluated by four trained graders from the Doheny Eye Institute.

**Results:**

Corneal epithelium in dry eyes presents morphological changes such as areas of enlarged and irregular shaped cells. In comparison with controls, the density of superficial epithelial cells was decreased in both the NSDE (*P* < 0.05) and SSDE groups (*P* < 0.01); the density of the outer layer of wing cells was smaller but not significantly different in NSDE (*P* > 0.05), but was lower in the SSDE group (*P* < 0.01); the density of the inner layer of wing cells was decreased in both the NSDE (*P* < 0.05) and SSDE groups (*P* < 0.01) and the density of basal epithelial cells was lower in both the NSDE (*P* < 0.01) and SSDE groups (*P* = 0.01). For all cell counts, the interclass correlation coefficient showed good agreement between graders (ICC =0.75 to 0.93).

**Conclusions:**

IVCM represents a reliable technique for examining the corneal epithelial microstructural changes associated with dry eyes, as well as for objectively and reproducibly quantifying cell densities within all corneal epithelial layers.

## Introduction

Dry eye is a frequently encountered ocular surface disease, with a high prevalence in the adult population [1]. Patients affected with dry eyes do not produce enough tears or their quality of tears is poor [2]. Grittiness, burning sensation, dryness, scratchiness, soreness, itching, foreign body sensation, in conjunction with blurred vision, represent common symptoms associated with dry eyes, affecting the daily quality of life for these patients. Dry eye disease’s etiology and management challenge clinicians and researchers alike. Due to its rather complex etiology, it was defined by the International Dry Eye Workshop (2007) as a “multifactorial disease of the tears and ocular surface that results in symptoms of discomfort, visual disturbance and tear film instability with potential damage to the ocular surface. It is accompanied by increased osmolarity of the tear film and inflammation of the ocular surface” [3].

Developing new therapies for dry eyes represents a big challenge for ophthalmologists. Further investigation of the pathophysiologic mechanisms underlying this condition are underway, and in vivo laser scanning confocal microscopy (IVCM) has emerged as a suitable, relatively novel, minimally invasive tool for obtaining high-resolution images of the living ocular surface at the cellular level. One of the commercially available Heidelberg retina tomograph (HRT) has been changed into a high-resolution digital laser scanning microscope for the visualization of anterior segment of the eye, by adding the Rostock Cornea Module (RCM). The instrument provides regular illumination of all corneal structures as the epithelium, corneal nerves, keratocytes and endothelium. The hydraulic z-scan allows a precise shift of the focus through the cornea, allowing to take series of images for the evaluation of the corneal cells profile and 3D reconstruction of various corneal structures. This method has been described in detail by Stave et al. [4]. The use of IVCM provides a new approach to evaluating the microscopic morphology of the cornea, offering images with a resolution comparable with histologic examination. The procedure is quick, non-invasive, safe and repeatable. IVCM is a promising technique, not only for the diagnosis of dry eye, but potentially for stratifying patients for clinical trials.

Dry eye is recognized nowadays as a disturbance of the “Lacrimal Functional Unit”, comprising of the lacrimal glands, ocular surface (cornea, conjunctiva, and meibomian glands) and lids, and the nerves connecting them [5]. Disease or damage to any component of the unit may disrupt the homeostasis of the whole ocular surface, eventually destabilizing the tear film and leading to ocular surface disease, expressed as dry eye [5–7].

Aqueous tear-deficient dry eye (ADDE) has two major subclasses, Sjögren’s syndrome dry eye (SSDE) and non- Sjögren’s syndrome dry eye (NSDE).

Sjögren’s syndrome is an exocrinopathy in which the lacrimal and salivary glands, along with other organs, are targeted by an autoimmune process. These glands are infiltrated by activated T-cells, causing acinar and ductular cell death and hyposecretion of tears and saliva. Therefore, the dry eye in Sjögren’s syndrome is due to lacrimal hyposecretion and the accompanying characteristic inflammatory changes in the lacrimal gland, together with the presence of inflammatory mediators in the tears and within the conjunctiva [8].

Non Sjögren’s dry eye represents a form of aqueous tear-deficient dry eye caused by lacrimal dysfunction, where the systemic autoimmune characteristics of SSDE have been excluded. The most common form of NSDE is age-related dry eye.

Treatment for dry eyes is aimed towards restoring or maintaining the normal amount and quality of tears in the eye, in order to minimize dryness and related discomfort and to maintain eye health.

Multiple studies have assessed corneal changes induced by dry eye and related conditions by using in vivo confocal microscopy [9, 10]. While most of them demonstrated reduced cellular densities within various epithelial layers, there is variability among reported values, potentially due to inconsistency in methodology and lack of accuracy in identifying individual cells for an objective analysis.

Our study aimed to evaluate morphological alterations and quantify the density of cells within the corneal epithelial layers of normal controls, NSDE and SSDE using IVCM, and to assess the reliability and reproducibility of data acquired by grading using the methodology described herewithin.

## Methods

Consecutive subjects with non-Sjögren’s and Sjögren’s syndrome dry eyes, as well as age-matched normal controls were prospectively recruited from the cornea clinics (single physician OLL) of the Doheny-UCLA eye center in Pasadena, California. The stage of the disease was determined by the examining physician by using OSDI scores, Schirmer’s test and tear break-up time. Sixty-eight eyes clinically diagnosed with aqueous tear-deficient dry eye syndrome; 24 eyes of 12 patients (11 women, 1 man; average age, 59 ± 22 years) with NSDE, and 44 eyes of 22 patients (21 women, 1 man; average age, 58 ± 9 years) with SSDE met the inclusion criteria for the study. Their corneal epithelial features were evaluated and compared with those of 10 eyes of 7 healthy volunteers (6 women, 1 man, average age, 59 ± 13 years). Previously, sub-epithelial area’s parameters - epithelial dendritic cells and corneal nerves - were evaluated in the same study cohort [11]. The diagnostic criteria defined by the American-European consensus group criteria (including a focus score ≥ 1 on labial salivary gland, or the presence of anti-SSA or anti-SSB antibodies) was considered for the diagnosis of Sjögren’s syndrome [12]. None of the patients with dry eyes or the normal subjects used topical or systemic NSAIDs (nonsteroidal anti-inflammatory drugs) or corticosteroids at the time of examination. The subjects in the control group had no history of eye disease and were free of any ocular symptoms or abnormality of the corneal epithelium. Normal subjects who had undergone any ocular surgery and those who wore contact-lenses were excluded. This study was performed with the approval of the Institutional Review Board/Ethics Committee of the University of California Los Angeles, and was conducted in a controlled, single-masked fashion. Signed written informed consent was obtained from all patients and healthy controls after a detailed explanation of the nature and purpose of the study prior to imaging. The study complied with the Health Insurance Portability and Accountability Act and adhered to the tenets of the Declaration of Helsinki.

Central corneal images were obtained for all subjects with a Heidelberg Retina Tomograph 3 with the Rostock Cornea Module (HRT III RCM, Heidelberg Engineering GmbH, Dossenheim, Germany) as previously described [13].

A total of 3–6 sequence/volume scans were taken from the center of each cornea, focusing on all corneal layers: superficial, intermediate/wing (“outer wing layer”, immediately beneath the superficial epithelial layer, and “inner wing layer”, immediately above the basal epithelial layer) and basal epithelial layers. At least three representative images of each epithelial layer of interest were selected for analysis for each eye, considering criteria such as cells clearly visible, whole image in the same layer, best focus and good contrast.

Four independent masked observers (the identity of subjects imaged with confocal microscopy as well as their medical diagnosis were masked) analyzed the images with respect to density of cells within the superficial, wing and basal epithelial layers. Two independent masked observers analyzed the confocal images with respect to characteristics and density of superficial and basal cells, and two other masked observers analyzed the confocal images with respect to characteristics and density of outer and inner wing cells. The cell density in each visual field of 160,000 μm^2^ was determined using the semi-automated cell count analysis software included with the instrument and was recorded as cells per square millimeter. The epithelial cells are manually and individually identified by the grader, while the instrument’s software automatically calculates the cell density. To quantify the density of superficial epithelial cells, all cells within a whole frame of 160,000 μm^2^ were counted. To obtain a standard area measurement for wing and basal cells (smaller and more dense), a ruler was placed on the screen and the cursor was used to draw a 7 cm line diagonally along the ruler, giving an area within the field of view between 19,000 μm^2^ and 20,000 μm^2^ and more than 100 cells counted per frame.

Statistical data analysis was performed using commercial software (Statistical Package for Social Science version 20.0, SPSS, Inc., Armonk, NY); and MedCalc version 12 (MedCalc software bvba; Mariakerke, Belgium). Summary data are reported as mean ± SEM. Data distribution was determined using the Kolmogorov-Smirnov test. Statistical comparisons of the mean values among groups were performed using ANOVA, together with a post-hoc Bonferroni test when necessary. *P* values less than 0.05 were considered statistically significant. Intraclass correlation coefficients and Bland-Altman plots were used to assess intergrader reproducibility.

## Results

Seventy-eight eyes were analyzed by four independent graders from Doheny Eye Institute. Demographic data, OSDI scores and Schirmer’s test scores (with anesthesia and without nasal mucosa stimulation – for evaluating the basal tear secretion) for all the participants to our study are summarized in Table 1.

**Table 1:** Demographic Data, Schirmer’s test and OSDI scores of Normal Controls and Patients with non-Sjögren’s and Sjögren’s Syndrome Dry Eye

	*Controls*	NSDE	SSDE
No. of Patients	*7*	12	22
Mean age ± SD (yrs.)	*59.3±12.7*	58.9±22.4	57.5±8.6
Gender (male/female)	*1/6*	1/11	1/21
Schirmer’s test (mm)	*15.3±5.7*	7.2*±4.2*^a^	2.3*±4.3*^a^
OSDI score	*6.25±5.89*	37.8±10.59^a^	46.49±29.86^a^

*NSDE* non-Sjögren’s dry eye, *SSDE* Sjögren’s syndrome dry eye, *OSDI* Ocular Surface Disease Index

Values reported as mean ± SD

^a^Statistically significant (*P* < 0.05) compared with controls

**Fig. 1:**
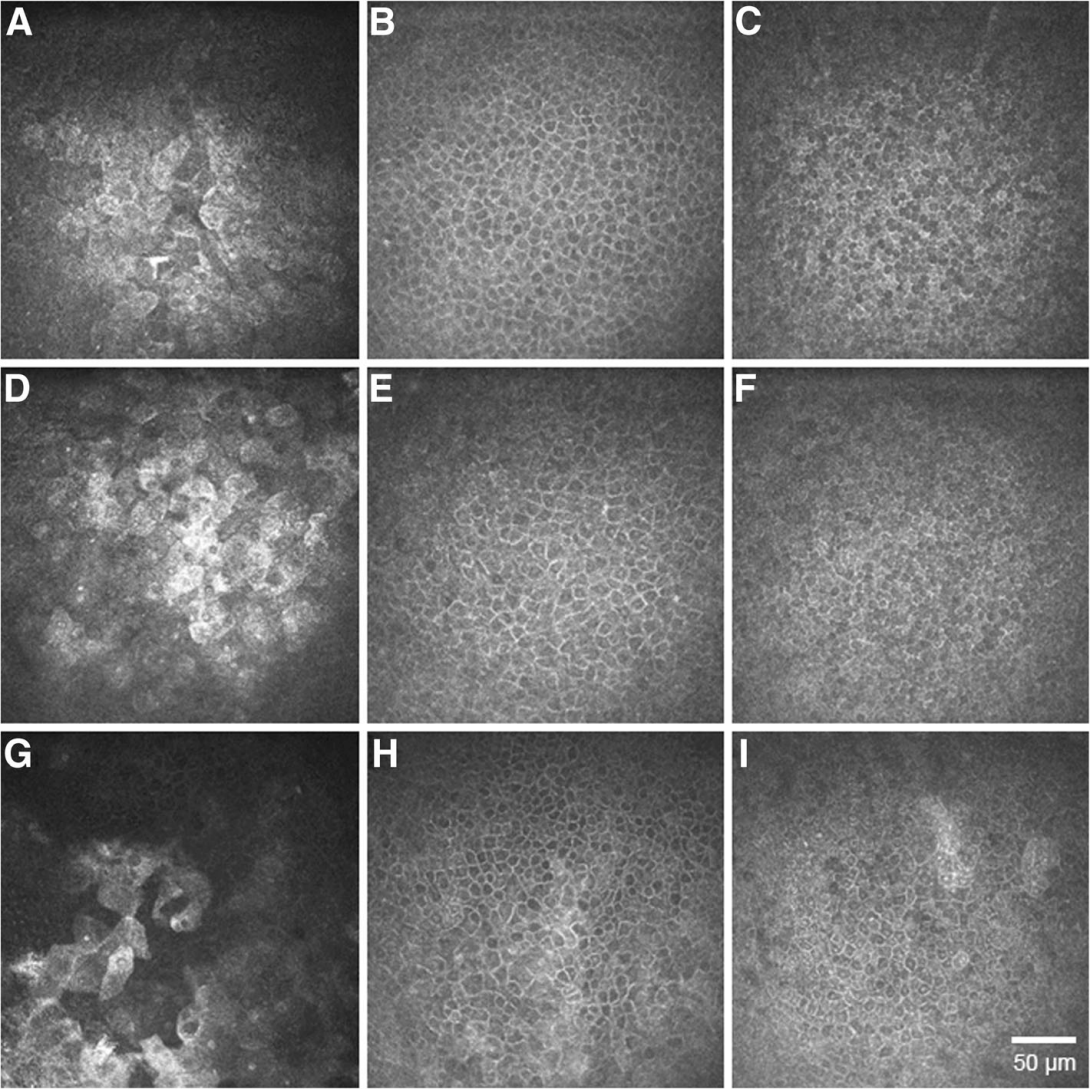
Representative images of corneal epithelial layers in normal eyes and the eyes of patients with non-Sjögren’s dry eye (NSDE) and Sjögren’s syndrome dry eye (SSDE). Normal controls: (**a**) normal superficial epithelial layer with hyper-reflective cells in the process of desquamation, presenting visible large nuclei; (**b**) wing layer with regular shape cells, presenting bright cell borders and dark cytoplasm; (**c**) basal epithelial layer with smaller cells presenting heterogeneous cytoplasmic reflectivity. Dry eye patients: (**d**, **e**, **f**) and (**g**, **h**, **i**) superficial, wing and basal epithelial layers for NSDE and SSDE respectively, showing superficial epithelial squamous metaplasia (enlarged and hyper-reflective cells), patchy alterations with enlarged and irregular cells within the wing and basal epithelial layers, thus lower cell density as compared to normal controls

Morphological changes were noted in the epithelial layers of dry-eye affected corneas, such as enlarged, highly hyper-reflective superficial epithelial cells, local alterations within the wing and basal epithelial layers with enlarged cells with irregular shape, and hyper-reflective cell borders, as illustrated in Fig. 1.

**Fig. 2:**
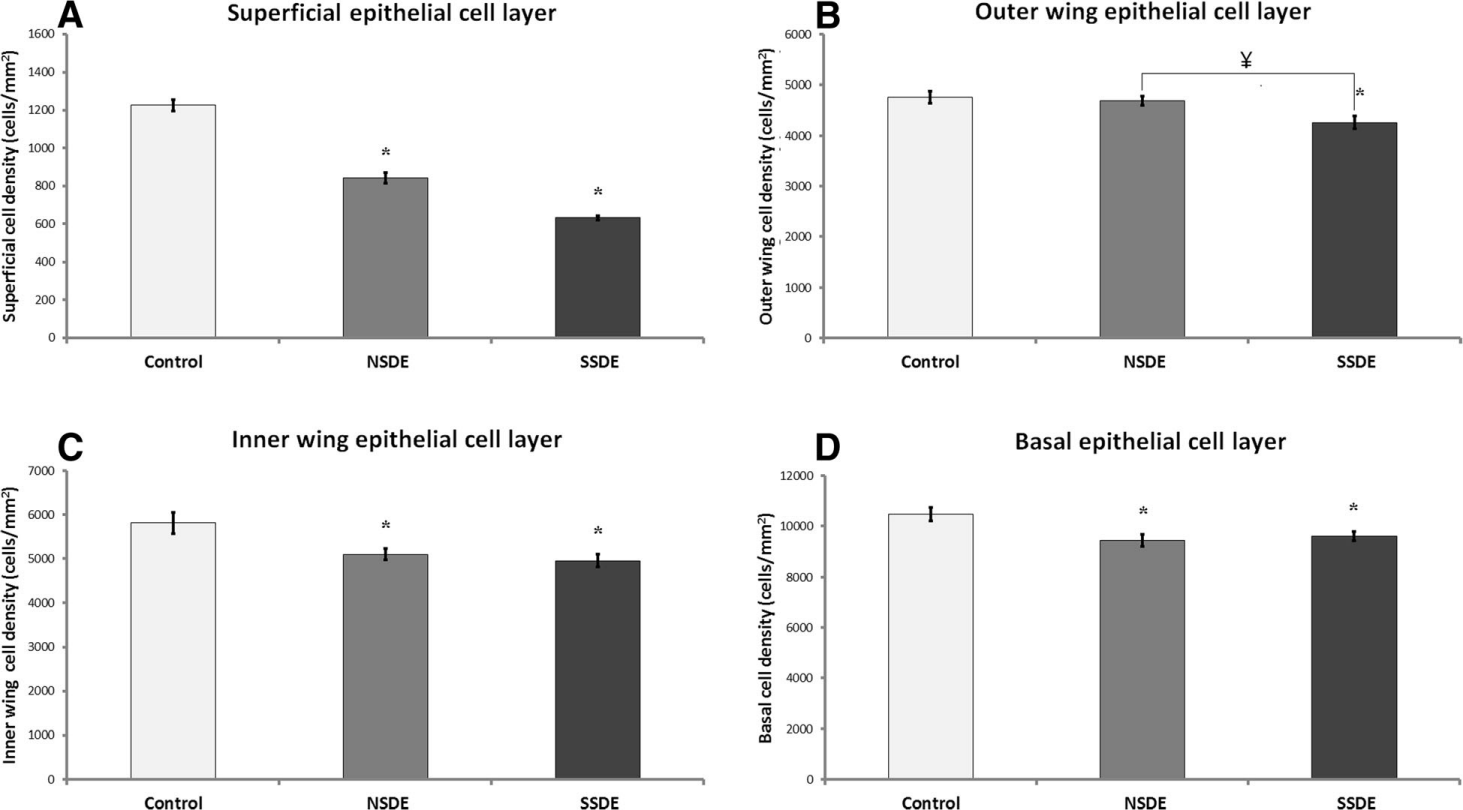
Corneal epithelial parameters in normal controls and dry eye patients. The density of superficial epithelial cells (**a**), outer wing cells (**b**), inner wing cells (**c**) and basal epithelial layer (**d**) was lower in the diseased eyes (non-Sjögren’s dry eye and Sjögren’s syndrome dry eye). Error bars represent standard errors from the mean (SEM). Statistical analysis by ANOVA, ^*^P < 0.05 compared to normal controls, ^¥^*P* < 0.05 comparing the NSDE and SSDE groups

The superficial, outer wing, inner wing and basal epithelial cell density in the central corneas were lower in dry eyes than in normal controls (*P* < 0.05, ANOVA) (Fig. 2). We demonstrated that superficial epithelial cell densities were significantly decreased in the NSDE (842.7 ± 137.3, *P* < 0.05) and SSDE groups (632.5 ± 69, *P* < 0.01) as compared to normal controls (1227 ± 96.6), showing no statistically significant difference between the NSDE and SSDE groups. Since the superficial epithelial cells could not be imaged in all eyes, we used for analysis 8 control eyes, 15 eyes with NSDE and 30 eyes with SSDE. The outer wing cell densities were decreased in the NSDE group, without reaching statistical significance (4686 ± 429.3, *P* = 0.6), and the SSDE group (4257 ± 839.2, *P* < 0.01) as compared to normal controls (4759 ± 380.5), showing a statistically significant difference between the NSDE and SSDE groups (*P* < 0.01). The inner wing cell densities were decreased in the NSDE (5097 ± 628, *P* < 0.05) and SSDE groups (4953 ± 943.7, *P* < 0.01) as compared to normal controls (5813 ± 772.7), showing no statistically significant difference between the NSDE and SSDE groups. The basal cell densities were decreased in both the NSDE (9429 ± 1132, *P* < 0.01) and SSDE groups (9600.6 ± 1179.9, *P* = 0.01) as compared to normal controls (10,479 ± 833.5), but with no statistically significant difference detected between the NSDE and SSDE groups.

**Fig. 3:**
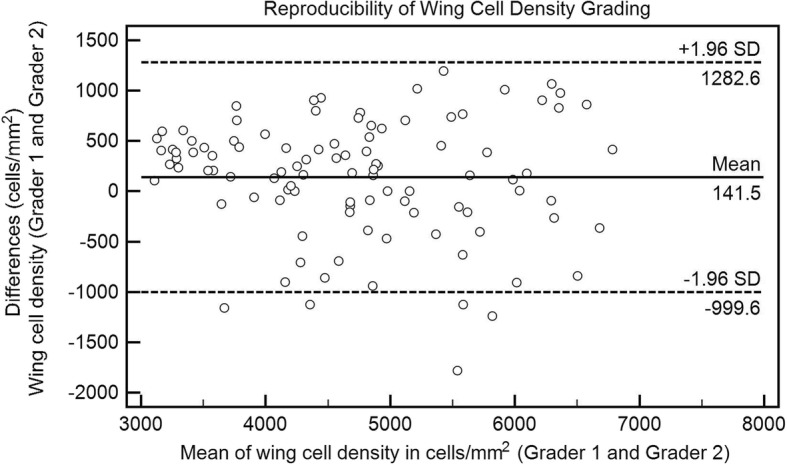
Bland-Altman plot illustrating the level of agreement between masked graders for determining the wing cell density. This assessment is based on masked grading of all the cases including normal controls, non-Sjögren’s dry eye and Sjögren’s syndrome dry eye patients. SD = standard deviation

The corneal epithelial cell densities for the three groups considered (control, NSDE and SSDE), as determined by two pairs of graders, showed good or excellent correlation, with the following interclass correlation coefficients for each of the 4 variables evaluated: 0.75 (95% confidence interval (CI), 0.01–0.94) for superficial epithelial cells; 0.85 (95% CI, 0.74–0.92) for outer wing cells; 0.93 (95% CI, 0.88–0.96) for inner wing cells; and 0.83 (95% CI, 0.38–0.96) for basal epithelial cells. The very high level of agreement between the two graders for evaluation of wing cell density (outer and inner wing cells) is illustrated by the Bland-Altman plot in Fig. 3.

## Discussion

This paper provides quantitative and qualitative analysis of the corneal epithelium in dry eye syndromes. We have shown morphological alterations of the superficial corneal epithelium, such as epithelial squamous metaplasia with an enhanced process of desquamation (many larger and hyper-reflective cells) and local alterations of the underlying epithelial surface (wing and basal layers) with enlarged cells presenting irregular shapes and visible nuclei. Initially, these patchy alterations and irregularities of the corneal epithelial cells, were described in patients with Sjögren’s syndrome [14], subsequently other studies highlighting these alterations of the corneal epithelium affected by dry eye disease [15–18].

We demonstrated that the overall cell densities were decreased in the epithelial layers of both NSDE- and SSDE-affected corneas, as compared to controls. The lowest density of cells within the epithelial layers, with the exception of the basal epithelial layer, was noticed in Sjögren’s syndrome-affected eyes, however, only the difference in outer wing cell density reaching statistical significance between the NSDE and SSDE cohorts. This might be explained by the relatively low number of participants in our study. Moreover, we have shown the reliability and reproducibility of cell counts obtained with the semi-automated cell counting software provided with the instrument, as illustrated by the levels of agreement between two independent graders. Although the HRT IIII RCM provides a small field of view of 400 × 400 μm (0.16 mm^2^), the reproducible investigation and quantification of the same area over time is possible by imaging the corneal apex. One shortcoming of using IVCM for the evaluation of corneal epithelium is the quantification of superficial epithelial cell density, since this layer is difficult to capture reliably, reducing the number of eyes to be analyzed.

Corneal epithelium acts as a natural barrier against potentially damaging agents from the environment and is very important for maintaining homeostasis of the ocular surface. Inflammation of the ocular surface has been shown to play an integral role in dry eye syndrome, with inflammatory mediators such as cytokines and proteolytic enzymes present over the entire ocular surface [19]. The hyper-osmolarity of the tear film associated with increased tear evaporation, may induce morphological and proinflammatory changes of the corneal epithelium [20–22], such as the local alterations we observed, eventually affecting the homeostasis of the whole ocular surface and further reducing the tear film quality and goblet cell function [23–25].

The microscopic evaluation of the corneal epithelium in dry eye patients (NSDE and SSDE) by IVCM demonstrates the significant morphological changes of the corneal epithelium that occur with dry eye. Furthermore, the cell density data shown in our study demonstrates significantly reduced epithelial cell density across all layers of the epithelium in dry eye syndrome affected eyes, possibly due to enhanced desquamation, inflammatory mediated apoptosis and impaired epithelial regeneration.

Previous studies reported reduced corneal thickness in dry eye patients [26, 27], as well as decreased density of superficial epithelial cells [23, 27–29] and decreased density of wing cells [23, 29], while the results reported for basal epithelial cells are conflicting: various authors have reported no change in the basal cell density of dry eyes [28, 29], increased basal cell density [27] or decreased basal cell density [23]. Our findings for superficial and intermediate epithelial cells are in agreement with previously published data, and the various results regarding the density of basal epithelial cells could be attributed to selection bias, possible different steps in the treatment (since all patients recruited for this study were on topical and/or systemic treatment at the time of investigation) as well as to the accuracy of cell count, since different studies provided different values for cellular densities within same corneal epithelial layers in both control and diseased eyes; the density of superficial epithelial cells in normal control subjects ranges from 1026 to 1398 cells/mm^2^ [17] to 1299–1758 cells/mm^2^ [16] and 536–947 cells/mm^2^ in primary Sjogrens dry eye to 785–1258 cells/mm^2^ in non-Sjogren’s dry eyes [16] while the basal cell density ranges from 5168 to 6348 cells/mm^2^ [16] to 11,307 ± 1876 cells/mm^2^ [18] in healthy subjects, and 5425–6044 cells/mm^2^ [16] to 9234 ± 1365 cells/mm^2^ [18] in dry eye affected corneas. These studies reported similar values for wing cell density in normal and dry eye affected cornea [17, 18]. The values we are reporting are in agreement with the cell count values determined by Erdelyi et al., [17] and Zhang et al., [18] both using the same confocal microscopy instrument as we did.The novelty of our investigation is that we have assessed the reproducibility of cell counts using the semi-automated software included with HRT III RCM in healthy controls and in patients with different degrees of involvement of the ocular surface, showing the accuracy and reliability of this measurement for various cell populations, emphasizing the best accuracy in cell count for wing cells, while using trained graders for such an assessment .

Petropoulos et al. demonstrated good repeatability for the manual assessment of all major corneal nerve fiber parameters, excepting nerve branch density, stating the heterogeneity in defining this parameter and suggest the necessity of using experienced observers or a method of automated analysis for this parameter [30].

Our study brings further insight into the extent of corneal epithelial damage induced by dry eye syndrome, demonstrating both qualitative and quantitative standardized and reproducible analysis of the corneal epithelia at the cellular level.

## Conclusions

Since IVCM can provide objective parameters for the evaluation of dry eye disease, it may be used as an adjunctive modality (in combination with other clinical measures) to evaluate and stratify patients for the purpose of diagnosis, prognostication and treatment decision making. Furthermore, herein lies the possibility of longitudinal comparison with implications for clinical research and clinical trials.
